# Antibacterial, Antifungal and Algicidal Activity of Phlorotannins, as Principal Biologically Active Components of Ten Species of Brown Algae

**DOI:** 10.3390/plants12040821

**Published:** 2023-02-12

**Authors:** Valeriya Lemesheva, Renata Islamova, Elena Stepchenkova, Aleksandr Shenfeld, Claudia Birkemeyer, Elena Tarakhovskaya

**Affiliations:** 1 Department of Plant Physiology and Biochemistry, Faculty of Biology, Saint Petersburg State University, Universitetskaya nab., 7/9, 199034 Saint Petersburg, Russia; 2 Department of Genetics and Biotechnology, Faculty of Biology, Saint Petersburg State University, Universitetskaya nab., 7/9, 199034 Saint Petersburg, Russia; 3 Vavilov Institute of General Genetics, Saint Petersburg Branch, Russian Academy of Science, Universitetskaya nab., 7/9, 199034 Saint Petersburg, Russia; 4 Faculty of Chemistry and Mineralogy, University of Leipzig, Linnestr. 3, 04103 Leipzig, Germany; 5 German Center for Integrative Biodiversity Research (iDiv), 04103 Leipzig, Germany

**Keywords:** phlorotannins, brown algae, toxicity, antibiotic activity, microalgae, bacteria, yeast

## Abstract

Marine seaweeds synthesize a plethora of bioactive metabolites, of which phlorotannins of brown algae currently attract special attention due to their high antibiotic and cytotoxic capacities. Here we measured the minimum inhibitory concentrations (MICs) of several semi-purified phlorotannin preparations of different origins and molecular composition using a set of model unicellular organisms, such as *Escherichia coli*, *Saccharomyces cerevisiae*, *Chlamydomonas reinhardtii,* etc. For the first time, MIC values were evaluated for phlorotannin-enriched extracts of brown algae of the orders Ectocarpales and Desmarestiales. Phlorotannin extracts of *Desmarestia aculeata*, *Fucus vesiculosus*, and *Ectocarpus siliculosus* showed the lowest MIC values against most of the treated organisms (4–25 μg/mL for bacteria and yeast). Analysis of the survival curves of *E. coli* showed that massive loss of cells started after 3–4 h of exposure. Microalgae were less susceptible to activity of phlorotannin extracts, with the highest MIC values (≥200 µg/mL) measured for *Chlorella vulgaris* cells. *D. aculeata*, *E. siliculosus*, and three fucalean algae accumulate considerable amounts (4–16% of dry weight) of phlorotannins with MIC values similar to those widely used antibiotics. As these species grow abundantly in polar and temperate seas and have considerable biomass, they may be regarded as promising sources of phlorotannins.

## 1. Introduction

**Figure 1:**
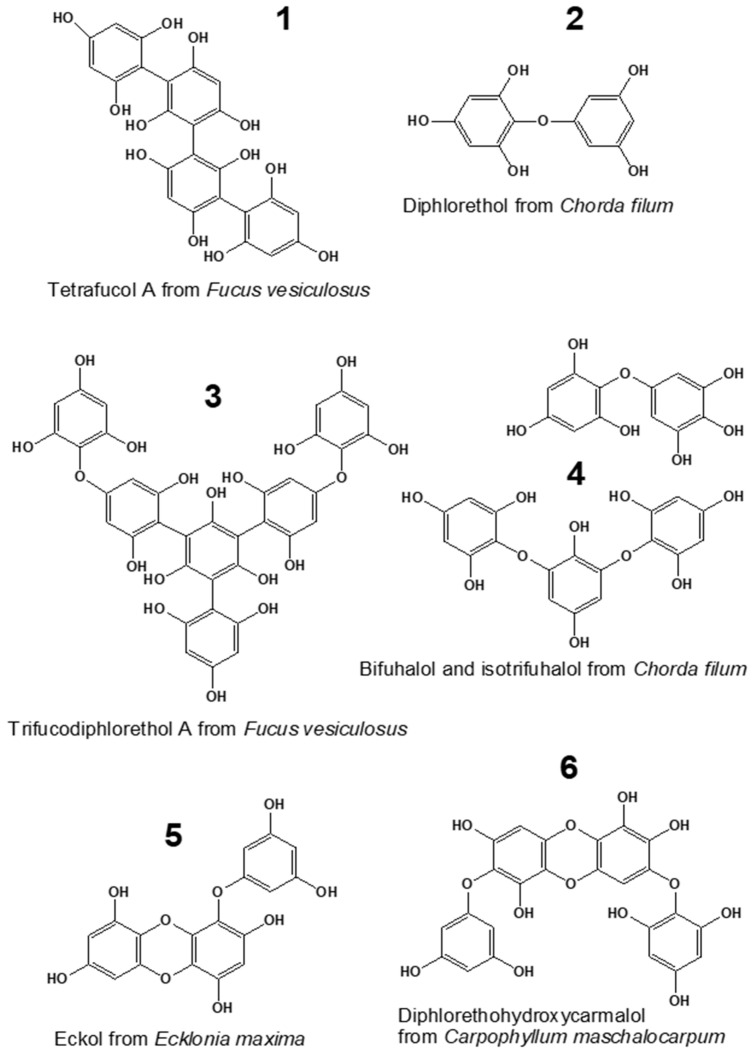
Compounds representing six major phlorotannin classes: fucols (**1**), phlorethols (**2**), fucophlorethols (**3**), fuhalols (**4**), eckols (**5**), and carmalols (**6**) [3,7,8].

Marine seaweeds are known as rich sources of specific bioactive substances, such as carrageenans, alginates, fucoidans, and phenols [1,2]. Phlorotannins, the unique phenolic compounds produced by brown algae, represent one of the most interesting classes of bioactive algal metabolites. These molecules are oligomers and polymers of phloroglucinol (1,3,5-trihydroxybenzene), widely varying in structure and degree of polymerization (DP). Among these compounds, up to six structural classes can be distinguished, depending on the bond type between the phloroglucinol moieties (aryl-aryl, ether, or combination of aryl and ether bonds) and the presence of additional hydroxyl groups (Figure 1) [3,4]. The molecular profiles of phlorotannins are very complex and species-specific. Considerable differences in polyphenol molecular structure and DP were shown even for closely related species of brown algae [5,6].

The content of phlorotannins in the cells of brown algae is currently studied in detail only for several species of the orders Laminariales, Fucales, and Dictyotales [9]. All these algae contain considerable amounts of polyphenols, varying from 0.5 to 30% of dry weight (DW), with the highest values reported from fucalean algae such as *Fucus*, *Ascophyllum* (Fucaceae), and *Carpophyllum* (Sargassaceae) [9,10,11]. In addition to interspecies variation, the content of phlorotannins also depends on some environmental and physiological factors, such as developmental stage, season, location, nutrient availability, and activity of biofoulers and grazers [12,13,14,15,16,17,18]. Studies of natural variations of phlorotannin content in brown algae are hampered by the absence of a universal protocol for the extraction of these metabolites. The diversity of established extraction techniques was comprehensively reviewed by Cassani et al. [19]. Mostly, extraction procedures include using some polar organic solvents with low boiling points (acetone, ethanol, methanol, ethyl acetate) and various physical treatments (ultrasound, microwaves, high temperature, high pressure, shaking). One of the most successful and robust protocols by Koivikko et al. [20,21] was employed for this study.

Phlorotannins have both primary and secondary metabolic roles in algal cells [22,23,24]. They are structural constituents of the cell walls of brown algae and contribute to the processes of embryogenesis [25,26]. Moreover, phlorotannins possess high antioxidative activity [27,28,29], scavenge heavy metals [30,31], and participate in wound-healing processes in brown algae [32]. The most pronounced and extensively studied function of phlorotannins is their contribution to the chemical defense of brown seaweeds against diverse deleterious microorganisms, biofoulers, and grazers [13,22,33,34,35,36]. This function is conferred by the considerable toxicity of these phenolic compounds, especially for unicellular organisms. Thus, phlorotannins have antibiotic effects against Gram-negative and Gram-positive bacteria, human pathogenic yeast and other microfungi, and microalgae [36,37,38,39,40]. In antimicrobial tests, several phlorotannin extracts demonstrated minimum inhibitory concentration (MIC) values close to those of widely used natural and synthetic antibiotics; moreover, some polyphenol preparations were effective even against antibiotic-resistant bacterial strains [40,41,42,43,44]. Currently, MIC values are measured precisely only for phlorotannins isolated from several fucalean and laminarialean species (*Ascophyllum nodosum*, *Eisenia bicyclis*, *Ecklonia cava*) [18,41,45]. Unfortunately, a considerable portion of the literature describing antibiotic effects of phlorotannins cannot be used for adequate comparison, as this data is either phenomenological or the authors used crude extracts without measuring phlorotannin content and thus could not attribute the biological effect to the amount of these specific metabolites [46]. Another factor contributing to data inconsistency is the use of incomparable methods (e.g., agar disk diffusion and broth microdilution) and different breakpoints for MIC or minimum bactericidal concentration evaluation [47].

Despite numerous studies of phlorotannin cytotoxicity, our understanding of the underlying mechanisms is still very limited. Traditionally, antibiotic effects of phlorotannins were regarded as the consequence of their ability to precipitate proteins, a general feature of phenolic compounds [48,49]. This implies that phlorotannin toxicity is non-specific. However, a considerable variation was shown for the antibiotic activity of polyphenols having different molecular profiles and isolated from different brown algae. Moreover, even phlorotannin molecules of the same structural class may differ in their biological effects. Thus, phlorofucofuroeckol-A isolated from *E. bicyclis* is especially toxic for microalgae and methicillin-resistant strains of *Staphylococcus aureus* [37,41]. At the same time, fucofuroeckol-A of the same origin is more effective against *Candida* species, streptomycin-resistant *Listeria monocytogenes,* and acne-related bacteria [43,44,50]. The antibiotic effect of phlorotannins may be not only bactericidal but also bacteriostatic [51]. In addition, a variety of specific responses was reported for eukaryotic unicellular organisms. e.g., phlorotannins inhibit the dimorphic transition in pathogenic yeast and reduce the buoyancy of microalgae [37,39]. Altogether, this data implies that the antibiotic activity of phlorotannins is complex and specific and that investigations on the molecular level are needed to reveal its mechanisms. Currently, practically focused investigations not considering the underlying mechanisms of phlorotannin toxicity are highly predominant among the studies of the biological effects of these metabolites (reviewed in: [52]). As a result, different data is obtained by different methods (often incomparable), mostly on pathogenic microorganisms, which are sometimes still poorly studied from a molecular and genetic perspective. To create a foundation for deeper mechanistic investigations in this field, a more systematic approach is needed. Phlorotannins of different origins should be extracted and quantified by the same protocol and then tested on the model objects of molecular biology and genetics, representing different taxa of unicellular organisms (e.g., *Escherichia coli* for prokaryotes, *Saccharomyces cerevisiae* for fungi, *Chlamydomonas reinhardtii* for microalgae, etc.). The benefits of this approach include: (1) the assessment of specific responses of organisms with different cell structures and surface composition to a variety of phlorotannin extracts from different algal species and (2) the possibility of using well-established, standardized methods on model organisms for further deeper molecular studies and revealing the mechanisms of phlorotannin action.

As the first and crucial step in every toxicological research is usually a precise determination of MIC values, the objective of our study is measuring MICs of several phlorotannin preparations of different origins using a set of model unicellular organisms. In addition, we analyzed the total phenolic content of the easily extractable intracellular fraction of phlorotannins in several brown algae representing different taxonomic groups.

## 2. Results

### 2.1. Phlorotannin Content and Chemical Characterization

**Figure 2:**
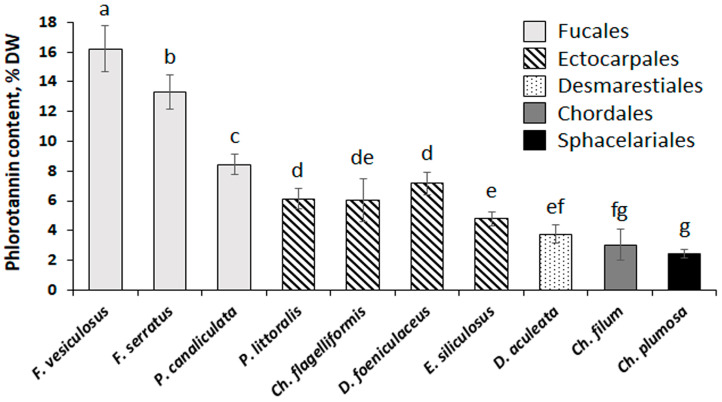
Content of phlorotannins in the algae representing five orders of Phaeophyceae (Fucales, Ectocarpales, Desmarestiales, Chordales, and Sphacelariales). Bars represent the mean ± SD (*n* = 6–12). Different letters indicate significant differences (*p* < 0.05, Student’s *t*-test).

The total content of phlorotannins in the studied brown algae varied from 2.4% DW (*Ch. plumosa*) to 16.2% DW (*F. vesiculosus*) (Figure 2). Representatives of the Fucales contained the highest amounts of phenolic substances, ectocarpalean species showed medium phlorotannin content (4.8–7.2% DW), and *Desmarestia*, *Chorda*, and *Chaetopteris* featured the lowest phlorotannin content (2.4–3.8% DW).

A comprehensive HPLC-MS analysis of three of the most biologically active extracts (*F. vesiculosus*, *F. serratus*, and *P. canaliculata*) carried out in our previous study [6] confirmed the effectivity of the extraction procedures, demonstrating that phlorotannins are the major constituents of the extracts and revealing specific phlorotannin profiles (Figures S1 and S2).

### 2.2. Minimum Inhibitory Concentrations of Phlorotannin Preparations

The toxicity of phlorotannin extracts varied considerably depending on both the source of polyphenols and the organism used as the test object (Table 1). Extracts of *D. aculeata* demonstrated the strongest antibiotic effect against all treated microorganisms, with MIC values ranging from 4 (for *S. cerevisiae*) to 300 (for *Chlorella*) µg/mL. Phlorotannins extracted from fucoid algae also had relatively low MIC values (5–20 µg/mL) for bacteria and yeast but were considerably less toxic for microalgae. Polyphenols of the Ectocarpales demonstrated high variation in their toxicity: extract of *E. siliculosus* had the lowest MIC values, comparable to those of fucoid algae, extracts *D. foeniculaceus* and *Ch. flagelliformis* showed moderate antibiotic activity, and extracts of *P. littoralis* were the least toxic against all the tested microorganisms.

Generally, microalgae were less susceptible to phlorotannins compared to bacteria and yeast (Table 1). *Chlorella* demonstrated the highest tolerance (all MIC values ≥ 200 µg/mL). The lowest MIC values of most phlorotannin preparations were registered in the experiments with yeast.

Phloroglucinol, the monomer of phlorotannins, at concentrations up to 1 mg/mL, was not toxic to any tested organism (Table 1).

### 2.3. Antibiotic Activity of Phlorotannin extracts against E. coli

**Figure 3:**
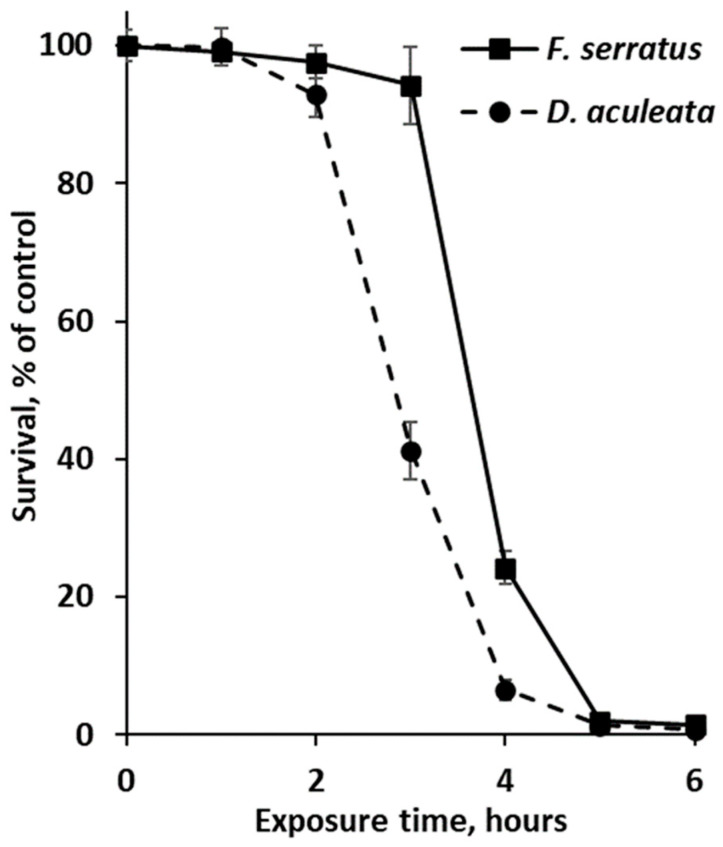
Survival curves of *Escherichia coli* cells in 0.9% NaCl solution supplemented with phlorotannin-enriched extracts of *Fucus serratus* and *Desmarestia aculeata* at the minimum inhibitory concentrations (20 and 5 μg/mL, respectively). Means are given with ±SD (*n* = 4).

As phlorotannin-enriched extracts of *Desmarestia* and fucoid algae showed the highest antibiotic activity, these preparations were additionally tested in experiments with non-growing and growing cultures of *E. coli*. The results of a time-kill assay with non-dividing bacterial cells (incubated in 0.9% NaCl solution) are shown in Figure 3. According to the survival curves, the toxic effect of phlorotannins was manifested after 3 (for *D. aculeata*) or 4 (for *F. serratus*) hours of exposure, and more than 98% of *E. coli* cells died after 5 h-long exposure to any of the phlorotannin preparations (Figure 3).

**Figure 4:**
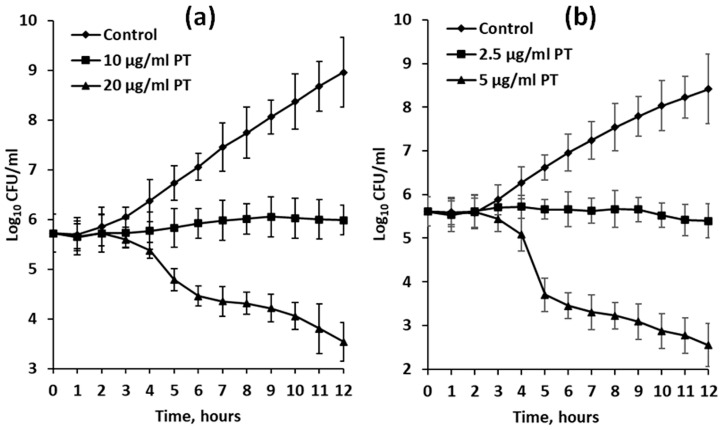
Growth curves of *Escherichia coli* cultures in VB medium (control) or VB medium supplemented with phlorotannin-enriched extracts (PT) of *Fucus serratus* (**a**) and *Desmarestia aculeata* (**b**). Means are given with ±SD (*n* = 4).

In the experiments with dividing bacterial cells, growth inhibition was visible after 3 h of exposure (Figure 4). When treated with phlorotannin extracts at half-MIC dosage (10 and 2.5 μg/mL for *F. serratus* and *D. aculeata*, respectively), cultures of *E. coli* stopped growing completely for the first 12 h (Figure 4). However, after 24 h of exposure, slight growth was visible (data not shown).

## 3. Discussion

Our results show that total phenolic content varies considerably in brown algae representing different taxonomic groups (Figure 2). As we measured this parameter in crude extracts, the values of total phenolic content may be used only as a proxy for the total content of phlorotannins in the algal thalli. However, phlorotannins are highly dominant in the phenolic profiles of brown algae, and it was shown that non-phenolic compounds contribute to less than 5% of Folin-Ciocalteu reactive substances [53]. This approach does not lead to a considerable overestimation of phlorotannin content. Relatively high amounts of polyphenols in the fucalean species (8–16% DW) correspond well to the literature data: thalli of *Fucus* spp., *P. canaliculata*, and *Ascophyllum nodosum* were shown to contain from 5 to 13% DW of phlorotannins, depending on location and season [31,33,54,55,56]. Different species of *Desmarestia* contain from 1.1 to 11.7% DW of phlorotannins [16,57,58,59], which is consistent with our results on *D. aculeata* (Figure 2). The other orders of brown algae are less studied from this perspective. Several ectocarpalean algae, including genera *Pylaiella* and *Dictyosiphon*, were reported to contain 0.23–1.7% DW of phlorotannins [60,61,62,63], which is less than in our study (Figure 2). The only studied representative of the order Sphacelariales (*Halopteris scoparia*) also contained fewer phlorotannins (0.16% DW) compared to our data on *Ch. plumosa* (Figure 2) [64]. Such discrepancy may be related to both the interspecies and geographical variation of phlorotannin content in brown algae. To our knowledge, our study is the first one focused on seaweeds inhabiting the Arctic Ocean. In contrast, all other available data on Ectocarpales and Sphacelariales was obtained from either tropical or temperate regions of the Atlantic and Pacific oceans [61,62,64]. Thus, we may suggest that the Arctic species of Ectocarpales and Sphacelariales are characterized by a higher content of phlorotannins. The possible reasons for this phenomenon warrant further investigation. We could not find any reports on phlorotannin content in *Chorda* or other species of the order Chordales. This small order combining 10 species was recently separated from the order Laminariales [65]. Given the phylogenetic similarity of these two taxonomic groups, we may compare phlorotannin content in *Chorda* with those in different laminarialean species. Among the Laminariales, the most studied are representatives of the families Laminariaceae containing 0.6–4% DW of phlorotannins, Lessoniaceae with 4–11% DW of phlorotannins, and Alariaceae containing 2–3% DW of phlorotannins [10,66,67]. Thus, we may conclude that phlorotannin content in *Ch. filum* is close to those of laminarialean algae (Figure 2).

Table 2 summarizes literature data on the toxicity of phlorotannins extracted from fucalean and laminarialean algae against different unicellular organisms. Analysis of this data shows a considerable discrepancy in the reported MIC values: while some studies showed relatively low (16–900 μg/mL) MICs [41,44,45,68], other publications presented values 2–3 orders of magnitude higher [38,39,50]. In the first group of studies, the tested phlorotannin preparations were carefully purified and separated chromatographically. As a result, authors worked with either individual molecules or mixtures containing several low-molecular-weight (DP 4–6) phlorotannins and quantified the biological activity of the extracts on their dry matter basis. This approach allows attributing the observed biological effects to particular phlorotannins. Another appropriate strategy of MIC assessment includes measuring the total phlorotannin content in purified or partially purified extracts without extensive chromatographic fractionation. Such an approach was used also in our study, as we tested semi-purified phlorotannin extracts containing the whole molecular profile. In this case, the biological activities may be attributed to the dominating phlorotannin species of the tested preparations or, possibly, particular active phlorotannin representatives. Though still very limited, literature data on the molecular composition of natural phlorotannins implies that brown algae do not synthesize the whole plethora of phlorotannins in equal quantities but tend to accumulate specific groups of phenolic molecules. Thus, *F. vesiculosus*, an alga in which phlorotannins demonstrated high antibiotic activities in our study, contains predominantly low-molecular-weight (DP 6–10) fucols and fucophlorethols, as was reported in our previous work [6]. The MIC values measured in our study using partially purified extracts with known total phlorotannin content (Table 1) are in the same range as data obtained using fractionated phlorotannins (Table 2 [41,44,45,68]), thus confirming that both methodical approaches produce comparable results. Meanwhile, we suppose that MIC values as high as 4000–31,300 μg/mL (Table 2 [38,39,50]) cannot be adequately compared to other literature data presented in Table 2 or to our own data (Table 1). In the studies reporting such high MIC values, crude or semi-purified algal extracts were used without measuring their total phlorotannin content. In this case, calculating the biological activity on the dry matter basis (as it was apparently conducted) may not be appropriate, as phlorotannins may contribute to a rather small portion of extract DW. Such methodology will inevitably lead to the overestimation of MICs, and we suppose this is one of the main reasons for the current inconsistency of literature data.

In general, though the literature describing the biological effects of phlorotannins is abundant, to date, only polyphenols of a limited number of algal species were tested for their MIC against bacteria and fungi (Table 2). Two laminarialean algae of the family Lessoniaceae (*E. bicyclis* and *E. cava*) gained particular interest due to the high toxicity of their phlorotannins. The dominating phlorotannin molecules of these algae are low-molecular-weight eckols (Figure 1), and the lowest MIC values against bacteria (16–32 μg/mL) were reported for fucofuroeckol-A from *E. bicyclis* [43,44]. In our study, phlorotannin-enriched extracts of *Chorda filum*, the species phylogenetically close to the Laminariales, demonstrated MIC values against Gram-negative bacteria, fungi, and microalgae in the same range as purified phlorotannin preparations of *E. bicyclis* and *E. cava* (Table 1 and Table 2). The analysis of the molecular composition of phlorotannins isolated from *Ch. filum* showed that they comprise low-molecular-weight fuhalols, isofuhalols, and phlorethols, with bifuhalol as the dominant compound (Figure 1) [8]. The other taxonomic group of brown algae accumulating phlorotannins with relatively low MIC values in toxicity tests is the order of Fucales. Among these algae, the lowest MICs against *E. coli* (25–50 μg/mL) were reported for extracts of *A. nodosum* [51]. We measured very close MIC values for three other fucalean algae, *Fucus vesiculosus*, *F. serratus,* and *Pelvetia canaliculata*, with extracts of *F. vesiculosus* being the most toxic (Table 1). In this case, we may attribute the toxic effect to low-molecular-weight fucols and fucophlorethols (Figure 1), which preferably accumulate in the cells of fucalean algae and are especially abundant in *F. vesiculosus*, according to our previously published data (Figure S1) [6].

Compared to polyphenols of laminarealen and fucalean algae, phlorotannins of the Desmarestiales, Ectocarpales, and Sphacelariales are much less studied. It was shown that phlorotannin extracts of Antarctic desmarestialean species, *Desmarestia anceps,* and *Phaeurus antarcticus*, had a relatively weak antibiotic effect against four marine bacterial strains and a stronger effect against several fouling diatom algae [75]. However, the MIC values of these phlorotannin preparations have not been measured, and we cannot directly compare these data to our results. In the present study, extracts of *D. aculeata* demonstrated the lowest MIC values against most of the tested organisms (Table 1). Unfortunately, the molecular profiles of phlorotannins of the Desmarestiales are still not studied, and thus further investigations are needed to reveal which phlorotannin species might confer such high toxicity.

Toxicological studies of phlorotannins isolated from Ectocarpales are also very scarce. We found only one research study reporting a considerable algicidal activity of the extracts of *Adenocystis utricularis* (Adenocystaceae) against marine fouling diatoms [75]. Unlike preparations of three fucalean algae having similar MIC values in our study, phlorotannin-enriched extracts of four tested ectocarpalean species demonstrated a wide variation in toxicity, with MIC values differing up to 40 times (Table 1). Ectocarpales is the largest order of brown algae, comprising several subordinate taxa of questionable position [76]. As a result, considerable differences in the biochemical composition may characterize the species belonging to this order. Indeed, the study of McInnes et al. [77] showed that representatives of different families of ectocarpalean algae preferentially synthesize phlorotannins of different classes. Thus, *Dictyosiphon foeniculaceus* and *Chordaria flagelliformis* (Chordariaceae) contain polyphenols with ether-linked phloroglucinol units with a high degree of hydroxylation (fuhalols), whereas *Pylaiella littoralis* (Acinetosporaceae) synthesizes almost exclusively phlorotannins with aryl linkages (fucols). Though the mentioned study was focused on high-molecular-weight polyphenols, the authors noted that all available data indicated a structural similarity of low- and high-molecular-weight phlorotannins of the same origin [77]. Combining this information with our data on the same three species (Table 1), we may suggest that fuhalol-enriched phlorotannin mixtures are generally more toxic compared to fucol-enriched ones, most probably due to the higher proportion of hydroxyl groups, in particular in ortho-position. It was shown that the antibiotic activity of polyphenols of higher plants (hydrolyzable and condensed tannins) depends on the relative content of the ortho-phenolic hydroxyl groups, as these groups mostly confer the affinity to bacterial surface proteins [78,79]. Unfortunately, the composition of phlorotannins of *Ectocarpus* is still not studied, though given their high antibiotic activity (Table 1), such an investigation would be in high demand.

The phlorotannin-enriched extract of the only representative of the order Sphacelariales used in this study (*Chaetopteris plumosa*) showed moderate toxicity comparable to that of some ectocarpalean species (*D. foeniculaceus*, *Ch. flagelliformis*) (Table 1). This result differs dramatically from data in the study by Lopes et al. [38], who reported MIC values of more than 30 mg/mL for extracts of three other sphacelarialean species (*Cladostephus spongiosus*, *Halopteris filicina*, *H. scoparia*). We may suggest two possible reasons for this discrepancy. First, sphacelarialean algae may differ considerably in both structure and biological activity of their phlorotannins, as shown for the Ectocarpales. Then, data inconsistency may be a result of different methods of MIC calculation, as discussed above.

According to our data, we may select five brown algal species (*F. vesiculosus*, *F. serratus*, *P. canaliculata*, *D. aculeata*, and *E. siliculosus*) demonstrating MIC values similar to those of widely used antibiotics, fungicides and algicides. For comparison, MIC of tetracycline, ampicillin, amoxicillin, and ceftiofur is 0.5–128 μg/mL for different isolates and strains of *E. coli* [80,81], MIC of fluconazole and amphotericin B towards different isolates of *S. cerevisiae* are 0.25–8 μg/mL [82], and MIC of diuron and atrazine for different species of *Chlamydomonas* and *Chlorella* are 2–11 μg/mL [83]. Moreover, even half-MIC dosage of the most active phlorotannin preparations tested in our study, though it did not kill bacterial cells, produced a prolonged bacteriostatic effect (Figure 4). While fucalean algae have already been reported as sources of highly toxic phlorotannins [51], MICs of *D. aculeata* and *E. siliculosus* were determined here for the first time.

In studies using semi-purified extracts containing the whole phlorotannin profile, it is desirable to minimize the potential interference of other biologically active compounds. Among the brown algal constituents extractable by organic polar solvents and occurring in the cells at concentrations comparable to those of phlorotannins, are photosynthetic pigments, such as chlorophyll *a* and fucoxanthin, and storage carbohydrates of low molecular weight, such as mannitol and volemitol. Multi-step washing with dichloromethane provided the elimination of lipids and pigments from our extracts. Cerantola et al. [84] and Audibert et al. [85], using similar protocols of phlorotannin extraction with polar organic solvents, reported that mannitol was still a major contaminant after the successive liquid-liquid separation with ethyl acetate. *Pelvetia* extracts may also contain some volemitol, a specific carbohydrate of this species [86]. However, both mannitol and volemitol are not toxic and, thus, are not expected to inhibit the growth of microorganisms to the observed extent when applied in concentrations comparable to MIC values measured in our study (Table 1). Moreover, our chromatograms of fucoid algae showed that apart from clear and abundant phlorotannin-related signals, any signal background was at least more than factor ten lower (Figure S2), so it can be reasoned that no other abundant compound could be detected in the used extracts during our earlier LC-MS analyses [6]. Thus, we reasonably suggest that the observed toxic effects of studied algal extracts may be attributed mainly to phlorotannins. However, further studies are underway to characterize the extracts with the most interesting activity in more detail.

To date, information about the mechanisms of phlorotannin toxicity is still very limited. As the relatively large hydrophilic molecules of phlorotannins cannot penetrate the intact cell membrane, the cell surface structures are obviously their first targets, and all available literature data agrees that phlorotannins damage the plasma membrane, thus resembling the biological effects of condensed and hydrolyzable tannins of vascular plants [18,51,87]. It was reported that cells of *E. coli* treated with phlorotannins had an altered surface structure: electron microphotographs showed surface loosening and disorganization, accompanied by the formation of some electron-dense precipitated deposits. The same effect, though to a lesser extent, was produced by hydrolyzable and condensed tannins of *Rhus semialata* and *Schinopsis balansae* [51]. Measurements of electrical conductivity in the suspensions of *E. coli*, *Salmonella agona*, *Vibrio parahaemolyticus*, and *Streptococcus suis* showed that phlorotannins caused a considerable increase in cell membrane permeability after 2–6 h of exposure [18,74]. This is consistent with our data on survival and growth curves of *E. coli* exposed to phlorotannins (Figure 3 and Figure 4). The increase in electrical conductivity in the bacterial suspensions is accompanied by leakage of intracellular and periplasmic proteins and a decrease in ATP content, which suggests the inhibition of oxidative phosphorylation, another membrane-associated process [18,74]. The inhibition of oxidative phosphorylation was also reported for the bacteria exposed to the tannins of vascular plants [88]. The responses of eukaryotic cells to phlorotannin treatment also mainly include alterations of surface structures. Phlorotannins of *Gongolaria* spp. reduced the ergosterol content in the plasma membrane of the yeast *Candida albicans* and dermatophyte fungus *Trichophyton rubrum*, thus compromising membrane integrity, and polyphenols of *Fucus spiralis* reduced the amount of chitin in the cell walls of *T. rubrum*. Moreover, these phlorotannin preparations inhibited adherence of *C. albicans* to epithelial cells [39].

Most probably, the first step in the progress of the reported damage to cell surface structures is the precipitation of surface-exposed proteins by phlorotannin oligomers. The ability of phlorotannins to precipitate proteins is higher compared to that of the tannins of terrestrial plants. Due to their susceptibility to spontaneous oxidation, phlorotannins, under natural conditions, cannot only form hydrogen bonds and non-polar interactions with proteins but also precipitate them via covalent bonds [48]. It was shown that the type and strength of phlorotannin-protein interactions considerably depend on the molecular structure of both phlorotannins and target proteins as well as on their proportion [48]. Thus, the most reactive phlorotannin species should be the ether-linked molecules that have ortho-substituted hydroxyl groups, making them prone to oxidation, and the most susceptible cells—those having the highest content of functional proteins exposed to the surface. This is consistent with our data on the MIC values, which were lowest for *E. coli* and yeast (Table 1). The outer membrane of *E. coli* consists of about 50% of proteins, playing key roles in the control of membrane permeability and stress resistance [89,90]. Moreover, the outer membrane proteins interact with peptidoglycans, the major constituents of the *E. coli* cell wall, which also may be targeted for phlorotannins [90]. The cell wall of *S. cerevisiae* also has a high content of specific proteins (about 15% of the wall constituents). In particular, a set of mannoproteins linked to the cell wall polysaccharides forms the outer layer of the wall, the first structure facing the phlorotannin impact [91,92]. Mannoproteins of *S. cerevisiae* are also known to interact readily with tannins of vascular plants, and this precipitation reaction is widely exploited in wineries (*e.g.*, [93]). Apparently, the resistance of the chlorococcalean microalga *Chlorella vulgaris* to phlorotannin treatment (Table 1) is also enabled by the specific features of the surface structure. Cell walls of many representatives of the Chlorococcales, including *Chlorella* spp., are extremely thick, robust, and chemically resistant [94]. Notably, among the Chlorococcales, the cell walls of *Chlorella* spp. have the lowest protein content (1.7–4.5%). The major constituents of the walls of *Ch. vulgaris* are neutral sugars, gluconic acids, and glucosamine [95].

According to the comprehensive study of Stern et al. [48], phlorotannins interact with proteins so fast that precipitation resulting in loss of protein functionality takes no more than several minutes. At the same time, analysis of the time-kill curves shows that bacterial cells exposed to phlorotannin-enriched extracts maintain viability for 2–3 h (Figure 3). This data suggests that though protein precipitation may be a crucial and, obviously, the first step in the phlorotannin action, it is accompanied by some other, more prolonged pathologic processes, presumably triggered by the phlorotannin entry into the cells through the damaged membranes.

## 4. Materials and Methods

### 4.1. Algal Material Collection

Brown algae (*Fucus vesiculosus* L., *F. serratus* L., *Pelvetia canaliculata* (L.) Dcne and Thur., *Pylaiella littoralis* (L.) Kjellm., *Chordaria flagelliformis* (O. F. Müll.) C. Ag., *Dictyosiphon foeniculaceus* (Huds.) Grev., *Ectocarpus siliculosus* (Dillw.) Lyngb., *Desmarestia aculeata* (L.) J. V. Lamour., *Chorda filum* (L.) Stackh., and *Chaetopteris plumosa* (Lyngb.) Kütz.) were collected in the Keret Archipelago (Kandalaksha Bay, White Sea; 66°17′28.76″ N 33°40′03.46″ E) in July–August 2020–2022. All algal species are named according to AlgaeBase [67]. Mature thalli were collected from the typical habitats of each species, cleaned from the epiphytes, rinsed with distilled water, carefully wiped with filter paper, and then immediately used for phlorotannin extraction or frozen and kept at –80 °C.

### 4.2. Phlorotannin Extraction

Phlorotannin extraction was carried out based on the standard protocol of Koivikko et al. [20,21]. For analysis of the total content of intracellular phlorotannins in the algal thalli, 20 mg of fresh algal material was poured with acetone:water (70:30, *v/v*) mixture, ground with mortar and pestle and left soaking in 1 mL aqueous acetone for one hour to extract intracellular phenolics. Then, the extract was centrifuged (5000× *g*, 10 min), the supernatant was transferred to another tube, and the pellet was re-extracted with another 1 mL of aqueous acetone. The supernatants of five extraction rounds were combined.

For toxicity tests, concentrated and semi-purified extracts were used. Samples of 1–2 g frozen algal material were homogenized using a cryogenic laboratory mill Freezer/Mill 6870 (SPEX SamplePrep, Metuchen, NJ, USA, Germany), transferred to the falcon tubes, poured with 10 mL of acetone:water (70:30, *v/v*) and left soaking for one hour. Then, each extract was centrifuged (5000× *g*, 10 min), the supernatant was transferred to another tube, and the pellet was re-extracted with another 10 mL of aqueous acetone. The supernatants of five extraction rounds were combined, and acetone was evaporated in a speed vac (CentriVap vacuum concentrator system, Labconco, Kansas City, MO, USA). Then the extracts were defatted three times, partitioning against dichloromethane (1:1, *v/v*), and phlorotannins were extracted by five successive portions of ethyl acetate (1:1, *v/v*). Ethyl acetate extracts were dried and resuspended in 1 mL water.

A comprehensive HPLC-MS analysis of three exemplary extracts (*F. vesiculosus*, *F. serratus*, and *P. canaliculata*) was carried out in our previous study [6], where we confirmed that phlorotannins are the principal constituents of the extracts and showed their specific molecular profiles. Koivikko et al. used a similar protocol of phlorotannin extraction and did not detect any major constituents besides phlorotannins by a combination of HPLC with UV- and MS-detection and NMR-analysis [21]. Improving chemical analysis to further and better characterize the phlorotannin profile of more species is an ongoing objective in our current research.

### 4.3. Analysis of Phlorotannin Content

A modification of the Folin–Ciocalteu micro-method was used to measure the total phlorotannin content in the crude and purified extracts [96]. Phloroglucinol (Sigma-Aldrich 79330) was used as the standard. The reaction mixture containing 0.3 mL of sample, 0.3 mL of Folin reagent, and 2.4 mL of 5% (*w/v*) aqueous Na_2_CO_3_ was incubated for 20 min at 45 °C, and then the absorbance was measured at 750 nm using a SPEKOL 1300 spectrophotometer (Analytik Jena AG, Jena, Germany).

### 4.4. Test Organisms and Growth Media

The following unicellular organisms were used for the bioassays: Gram-negative bacteria *Escherichia coli* strain KA796 (*ara thi D*(*pro-lac*)) [97], ascomycete yeast *Saccharomyces cerevisiae* haploid strain LAN201-ura3Δ (*MATa ade5-1 ura3Δ lys2-Tn5-13 trp1-289 his7-2 leu2-3,112*) [98], euglenoid protist *Euglena gracilis* Klebs strain Z, and two green microalgae, *Chlamydomonas reinhardtii* P. A. Dang. strain CC-124 and *Chlorella vulgaris* Beijer. strain Pringsheim. *E. coli*, S. *cerevisiae,* and *Ch. reinhardtii* were obtained from the collection of the Department of Genetics and Biotechnology, St. Petersburg State University. *E. gracilis* and *Ch. vulgaris* were obtained from the Resource Centre “Culture Collection of Microorganisms” of St. Petersburg State University.

*E. coli* was cultured in a complete LB medium [99] or minimal Vogel-Bonner medium containing proline (25 mg/L) [100]. *S. cerevisiae* was grown in complete YPD medium or minimal SD media (Yeast Nitrogen Base 6.7 g/L; glucose 2%) containing adenine (20 mg/L), uracil (20 mg/L), lysine (30 mg/L), tryptophan (20 mg/L), histidine (20 mg/L), and leucine (60 mg/L) [101]. All treatments with phlorotannin extracts were carried out in minimal media. Complete media were used to obtain stock cultures of microorganisms and for growing bacteria and yeast after phlorotannin exposure. *Euglena*, *Chlamydomonas*, and *Chlorella* were grown phototrophycally under continuous light (50 μM photons/m^2^c) in the mineral Cramer-Myers, TAP, and BBM media, respectively [102,103,104].

### 4.5. Measurement of Minimum Inhibitory Concentrations

MIC values were determined as the lowest concentrations of tested phlorotannin extracts, completely inhibiting the growth of the microorganism cultures. In addition to phlorotannins isolated from brown algae, an aqueous solution of phloroglucinol was also tested. The broth dilution method was used for the MIC assays [105]. First, the stock solutions of phlorotannin extracts were made in the corresponding growth media, and serial 2-fold dilutions of the stock were prepared in 96-well microtiter plates (100 μL per well). Then, 100 μL aliquots of cell suspensions containing approximately 10^4^ cells/mL of bacteria and yeast, and 10^5^ cells/mL of microalgae, respectively, were inoculated into each well. The final concentration of phlorotannin extracts/phloroglucinol ranged from 1 to 1000 μg/mL; pure growth medium was used as a negative control. For *E. coli* and *S. cerevisiae*, the MIC values were visually determined after 24 or 48 h of incubation, respectively. For microalgae, the MIC values were estimated after 4 days of incubation by counting the cells in a hemocytometer.

### 4.6. Antibiotic Activity Assay

Two variants of bioassays were carried out for a more detailed study of the antibiotic effect of phlorotannins extracted from *F. serratus* and *D. aculeata* against *E. coli*.

Bactericidal activity was tested using a time-kill assay for non-dividing *E. coli* cells. An overnight culture of *E. coli* was diluted 4 × 10^5^ times with 0.9% NaCl solution supplemented with phlorotannin-enriched extracts at the minimum inhibitory concentrations (20 μg/mL for *F. serratus* extract and 5 μg/mL for *D. aculeata* extract). Pure NaCl solution was used as a negative control. Then, the cell suspensions were incubated at 37 °C for 6 h, and 50 μL aliquots were plated on LB media every hour. The plates were incubated overnight at 37 °C, and the viable colony-forming units (CFU) were counted.

To test both the bacteriostatic and bactericidal activity of phlorotannins for the growing microbial culture, the growth curves of *E. coli* were analyzed. Overnight culture of *E. coli* was diluted 4 × 10^5^ times with fresh media supplemented with phlorotannins at half-MIC and MIC (10–20 μg/mL for *F. serratus* extract and 2.5–5 μg/mL for *D. aculeata* extract). A pure growth medium was used as a negative control. Then, cell suspensions were incubated at 37 °C for 12 h, and 100 μL aliquots (diluted to contain ~400 CFU) were plated on LB media every hour. The plates were incubated overnight at 37 °C, and the number of viable CFU was counted.

### 4.7. Data Analysis

Measurements were performed with four (survival and growth curves) to twelve (total phenolic content) biological replicates. An individual seaweed organism was used to obtain each analyzed phlorotannin sample. Excel 2016 (Microsoft, Redmond, WA, USA), Photoshop CS2 (Adobe Inc., San Jose, CA, USA), and ChemDraw Ultra (CambridgeSoft, Cambridge, MA, USA) software was used for data processing and creating of figures. Where appropriate, data were tested for normality using the Kolmogorov–Smirnov test and for homogeneity of variance using the Levene test. Student’s t-test was used to confirm the significant differences between the means. All values are expressed as means and standard deviations.

## 5. Conclusions

In our study, we investigated MICs of several partially purified phlorotannin preparations of different origins using a set of unicellular model organisms: *E. coli*, *S. cerevisiae*, *Ch. reinhardtii*, *E. gracilis*, and *Ch. vulgaris*. For the first time, MIC values were evaluated for phlorotannin-enriched extracts of the brown algae of the orders Ectocarpales and Desmarestiales. Five of the tested algal extracts (those of *D. aculeata*, *E. siliculosus*, and three fucalean algae) demonstrated high toxicity with MIC values similar to those of widely used antibiotics. As all these species grow abundantly in polar and temperate seas and have considerable biomass and relatively high phlorotannin content, they may be regarded as promising sources of these valuable metabolites. Using common model organisms of molecular biology and genetics for the toxicological assays established the basis required for further mechanistic investigations of phlorotannin bioactivity. Certainly, one of the future imperatives of phlorotannin research should also be the production and availability of single phlorotannin standards for more systematic investigations of structure-activity relationships (SAR).

## Figures and Tables

**Table 1:** Minimum inhibitory concentrations (MIC, µg/mL) of phloroglucinol and phlorotannin-enriched extracts of ten brown algal species against the unicellular test organisms.

Tested Phlorotannin Extracts/Compounds	Test Organisms				
	*E. coli*	*S. cerevisiae*	*E. gracilis*	*Ch. reinhardtii*	*Ch. vulgaris*
*Chorda filum*	100	40	250	60	200
*Fucus vesiculosus*	10	5	100	150	500
*Fucus serratus*	20	10	150	100	400
*Pelvetia canaliculata*	20	10	100	130	400
*Desmarestia aculeata*	5	4	30	60	300
*Ectocarpus siliculosus*	25	25	100	60	500
*Chordaria flagelliformis*	70	150	400	300	900
*Dictyosiphon foeniculaceus*	300	300	400	150	800
*Pylaiella littoralis*	>1000	400	800	400	>1000
*Chaetopteris plumosa*	300	200	200	150	400
Phloroglucinol	>1000	>1000	>1000	>1000	>1000

**Table 2:** Minimum inhibitory concentration (MIC, μg/mL) of different phlorotannin preparations against bacteria, fungi, and microalgae according to available literature data. Algal species are named according to the current AlgaeBase classification [69].

Test Organisms		Brown Algae, Source of Phlorotannins	MIC Values	References
Laminariales				
Gram-positive bacteria	*Staphylococcus aureus* (methicillin-resistant strain)	*Ecklonia cava* ssp. *stolonifera*	500–600	[42]
		*E. cava* ssp. *kurome*	64–128	[68]
		*Eisenia bicyclis*	100–200	[45]
		*E. bicyclis*	128–256	[43]
		*E. bicyclis*	32–64	[41]
	*S. epidermidis*	*E. bicyclis*	64–128	[43]
	*Streptococcus pyogenes*	*E. cava* ssp. *kurome*	400	[45]
	*Bacillus cereus*	*E. cava* ssp. *kurome*	200–400	
	*B. subtilis*	*E. cava* ssp. *stolonifera*	600	[42]
		*E. cava* ssp. *stolonifera*	64	[68]
	*Listeria monocytogenes* (streptomycin-resistant strain)	*E. bicyclis*	16–256	[44]
	*Propionibacterium acnes*	*E. bicyclis*	32–256	[43]
		*E. cava*	39–312	[70]
	*Enterococcus faecalis*	*E. cava*	128	[71]
Gram-negative bacteria	*Acinetobacter* sp.	*E. cava* ssp. *stolonifera*	128	[68]
	*Escherichia coli*	*E. cava* ssp. *kurome*	200–400	[45]
		*E. cava* ssp. *stolonifera*	256	[68]
		*E. cava* ssp. *stolonifera*	500–900	[42]
	*Vibrio parahaemolyticus*	*E. cava* ssp. *stolonifera*	600	
		*E. cava* ssp. *kurome*	200	[45]
	*Campylobacter jejuni*		50	
	*C. fetus*		50	
	*Salmonella enteritidis*		200–800	
	*S. typhimurium*		200	
		*E. cava* ssp. *stolonifera*	500	[42]
			256	[68]
	*Pseudomonas aeruginosa*	*E. bicyclis*	>1024	[43]
	*Klebsiella pneumoniae*	*E. cava* ssp. *stolonifera*	600	[42]
			256	[68]
Algae	*Karenia mikimotoi*	*E. cava* ssp. *kurome*	100	[37]
	*Cochlodinium polykrikoides*			
	*Chattonella antiqua*			
Fungi	*Trichophyton rubrum*	*Ecklonia cava*	148 *	[72]
	*Candida albicans*	*E. bicyclis*	4000–8000 512–2048	[50,73]
	* Candida glabrata *	*E. bicyclis*	4000–8000	[50]
Fucales				
Gram-positive bacteria	*Streptococcus suis*	*Ascophyllum nodosum*	781	[18]
		*Fucus serratus*	3125	
	*Staphylococcus aureus*	*Fucus spiralis*	7800	[38]
		*Gongolaria nodicaulis*	7800	
		*Gongolaria usneoides*	15,600	
		*Sargassum vulgare*	31,300	
	*S. epidermidis*	*Fucus spiralis*	3900	
		*Gongolaria nodicaulis*	3900	
		*Gongolaria usneoides*	7800	
		*Sargassum vulgare*	7800	
	*Micrococcus luteus*	*Fucus spiralis*	2000	
		*Gongolaria nodicaulis*	15,600	
		*Gongolaria usneoides*	31,300	
		*Sargassum vulgare*	>31,300	
Gram-negative bacteria	*Escherichia coli*	*Ascophyllum nodosum*	781	[18]
		*Ascophyllum nodosum*	25–50	[51]
		*Fucus serratus*	3125	[18]
		*Fucus spiralis*	>31,300	[38]
		*Gongolaria nodicaulis*	>31,300	
		*Gongolaria usneoides*	>31,300	
		*Sargassum vulgare*	>31,300	
	* Salmonella typhimurium *	*Fucus spiralis*	>31,300	[38]
	* Salmonella agona *	*Ascophyllum nodosum*	1560	[18]
		*Fucus serratus*	3125	
	* Vibrio parahaemolyticus *	*Sargassum thunbergii*	900	[74]
	* Pseudomonas aeruginosa *	*Fucus spiralis*	31,300	[38]
		*Gongolaria nodicaulis*	31,300	
		*Gongolaria usneoides*	>31,300	
		*Sargassum vulgare*	>31,300	
Fungi		*Gongolaria usneoides*	31,300	[39]
		*Gongolaria nodicaulis*	15,600	
		*Gongolaria usneoides*	31,300	
	* Candida parapsilosis *	*Fucus spiralis*	>62,500	
		*Gongolaria nodicaulis*	62,500	
		*Gongolaria usneoides*	62,500	
	* Aspergillus species *	*Fucus spiralis*	>62,500	
		*Gongolaria nodicaulis*	>62,500	
		*Gongolaria usneoides*	>62,500	
	* Epidermophyton floccosum *	*Fucus spiralis*	7800	
		*Gongolaria nodicaulis*	3900	
		*Gongolaria usneoides*	15,600	
	* Trichophyton rubrum *	*Fucus spiralis*	3900	
		*Gongolaria nodicaulis*	3900	
		*Gongolaria usneoides*	7800	
	* T. mentagrophytes *	*Fucus spiralis*	15,600	
		*Gongolaria nodicaulis*	7800	

* Original value is 200 μM of purified dieckol (MW 742 g/mol), which corresponds to 148 μg/mL.

## Data Availability

The datasets generated and/or analyzed in this study are available from the corresponding author upon reasonable request.

## Supplementary Materials

The following supporting information can be downloaded at: https://www.mdpi.com/article/10.3390/plants12040821/s1, Figure S1: Relative contribution of eight major types of phlorotannin molecules to the total pool of phlorotannins in the brown algae *Fucus vesiculosus*, *F. serratus*, and *Pelvetia canaliculata*; Figure S2: HPLC-MS analysis of the phlorotannin-enriched extract of brown alga *Fucus vesiculosus*. Reference [6] is cited in the supplementary materials.
