# Supplementary material for: Antibacterial, Antifungal and Algicidal Activity of Phlorotannins, as Principal Biologically Active Components of Ten Species of Brown Algae
Source: Plants (Basel). 2023 Feb 12;12(4):821. doi: 10.3390/plants12040821 (PMC9966351; doi:10.3390/plants12040821)
Supplement: Supplementary file 1 [file plants-12-00821-s001.zip › plants-2050725-SI.pdf]

HPLC-ESI-MS profiling of phlorotannins extracted from *Fucus vesiculosus*, *F. serra-tus*, and *Pelvetia canaliculata*

In our earlier paper [6], we describe the chemical analysis of phlorotannin extracts used for this work by LC-MS on an Agilent 1100 HPLC system (Agilent, Waldbronn, Germany) coupled to mass spectrometry. Briefly, 50 µL phlorotannin extracts (n=5) were injected onto a reversed-phase column Gemini C18, 5 µm, 110 Å, 150 mm x 2 mm (Phenomenex, Aschaffenburg, Germany) and separated at a flow rate of 0.4 mL/min by gradient elution using 0.1 % formic acid in water (B) and acetonitrile (A) as eluents (time in min/%B): 0.0/100, 10.0/100, 20.0/70, 30.0/70, 40.0/0, 50.0/0. MS data was acquired on a Bruker Esquire 3000 Plus ESI ion trap mass spectrometer (Bruker Daltonics, Bremen, Germany) in negative ion mode at a nebulizer pressure of 40 psi, dry gas flow at 9 L/min and dry gas temperature of 365 °C with *m*/*z* 1000 as the target mass. Data evaluation was based on cumulative peak intensity in averaged mass spectra.

We identified several phlorotannin series in the extracts and showed that the total pool of intracellular phlorotannins of the fucoid algae contains eight major types of molecules: (1) fucols/phlorethols, F/P; (2) fuhalols, Fh; (3) acetylated fucols/phlorethols, ac F/P; (4) eckols/carmalols, E/C; (5) hydroxylated eckols/carmalols, OH E/C; (6) dihydroxylated eckols/carmalols, 2OH E/C; (7) acetylated hydroxyl eckols/carmalols, ac OH E/C; (8) acetylated benzodioxin-eckols/carmalols, ac Bd E/C.

Figure S1 shows the corresponding phlorotannin molecular profiles of three studied algal species. Despite the phylogenetic proximity of these seaweeds (all representing the family Fucaceae) they differ considerably in their phlorotannin profiles. While in the cells of *F. vesiculosus* aryl and ether-linked molecules of F/P-type are highly dominating, *F. ser-ratus* and, especially, *Pelvetia* have more “balanced” profiles with relatively high proportions of dibenzodioxin phlorotannins, such as hydroxylated and dihydroxylated E/C (Fig-ure S1). A more detailed analysis of brown algal phlorotannin profiles, including the intra-thallus distribution of phlorotannin molecules differing in their structure and polymeri-zation degree, is presented in our previous publication [6].

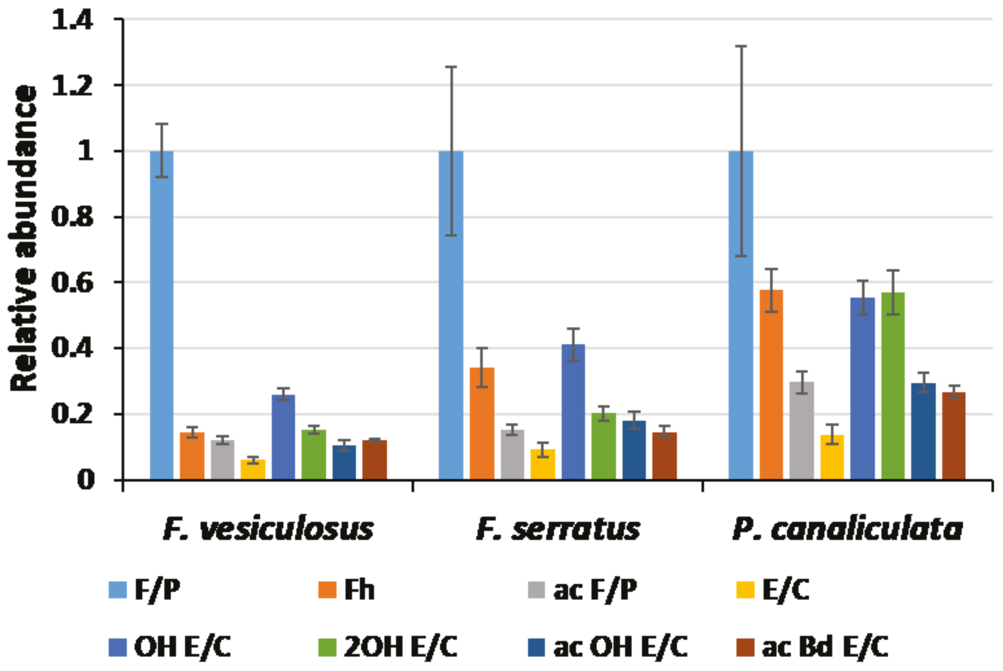

Figure S1. Relative contribution of eight major types of phlorotannin molecules to the total pool of phlorotannins in the brown algae *Fucus vesiculosus*, *F. serratus*, and *Pelvetia canaliculata*. Intensities of phlorotannin series-related MS-signals are normalized to the intensity of the dominating F/P molecules. **F/P** - fucols/phlorethols, **Fh** - fuhalols, **ac F/P** - acetylated fucols/phlorethols, **E/C** - eckols/carmalols, **OH E/C** - hydroxylated eckols/carmalols, **2OH E/C** - dihydroxylated eckols/carmalols, **ac OH E/C** - acetylated hydroxyl eckols/carmalols, **ac Bd E/C** - acetylated benzodioxin-eckols/carmalols.

HPLC-MS analysis clearly confirms that phlorotannins are the major constituents of the tested extracts, as data reveals distinct and abundant phlorotannin-related signals, whereas the signal background produced by co-extracted compounds is more than ten times lower (Figure S2).

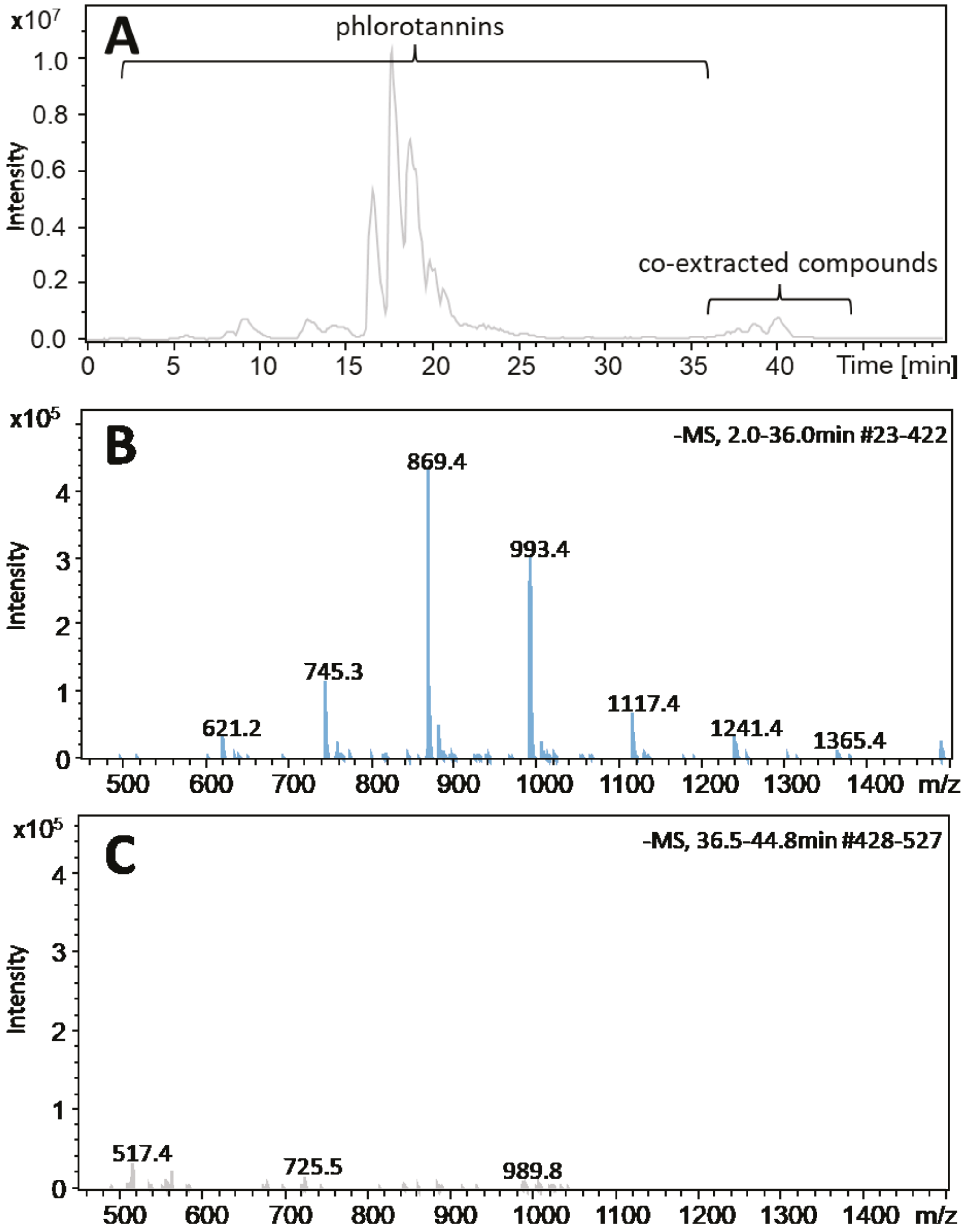

Figure S2. HPLC-MS analysis of the phlorotannin-enriched extract of brown alga *Fucus vesiculosus*. **A** - typical base peak chromatogram; **B** – MS-spectrum of the main phlorotannin profile (the most abundant signals refer to fucol/phloretol molecules with polymerization degree 5-11; **C** – MS-spectrum showing the signal background after the elution of phlorotannins.

References

6. Birkemeyer, C.; Lemesheva, V.; Billig, S.; Tarakhovskaya, E. Composition of intracellular and cell wall-bound phlorotannin fractions in fucoid algae indicates specific functions of these metabolites dependent on the chemical structure. *Metabolites*. **2020**, *10*, 369. https://doi.org/10.3390/metabo10090369
